# Clinical efficacy of optimized drug treatment for acute type A aortic dissection: insights from a multicenter retrospective cohort study

**DOI:** 10.1186/s40779-025-00638-8

**Published:** 2025-08-22

**Authors:** Shi-Pan Wang, Hai-Yang Li, Yin-Fan Zhu, Hao-Tian Wu, Dong-Jie Li, Yuan-Fei Zhao, Lu Dai, Wen-Jian Jiang, Hong-Jia Zhang

**Affiliations:** 1 Beijing Anzhen Hospital, Capital Medical University, Beijing, 100029 China; 2 Beijing Institute of Heart, Lung and Blood Vessel Diseases, Beijing, 100029 China; 3 Beijing Lab for Cardiovascular Precision Medicine, Beijing, 100029 China

**Keywords:** Multicenter study, Optimized drug treatment, Acute type A aortic dissection, Clinical prognosis

Dear Editor,

Acute aortic dissection (AAD) refers to the tearing of the aortic intima, with high-pressure blood flowing into the media. It can be classified into Stanford type A and type B according to whether the ascending aorta is involved. Acute type A aortic dissection (ATAAD) is a life-threatening cardiovascular disease with high mortality rates (approximately 50% and 1–2% per hour) within the first 48 h [1]. The most significant threat is that high-pressure blood flow may further damage the torn aortic wall, leading to aortic rupture and sudden death. ATAAD can also lead to a poor prognosis through complications such as malperfusion syndrome, which results from ischemic-hypoxic damage to organs supplied by branch vessels.

Current guideline recommends surgical procedures as the main treatment for ATAAD [2]. However, many patients cannot receive timely surgery due to financial constraints, concerns about surgical risks, or a lack of nearby capable medical centers, especially in developing countries. Although guideline suggests drug treatment immediately, including strict blood pressure control, heart rate management, and analgesia for ATAAD [3], they lack specific guidance on drug types and dosages, leading to a decrease in the compliance rate of the guidelines. Therefore, it becomes extremely important to engage in discussions and emphasize drug treatment approaches for ATAAD.

This multicenter retrospective observational cohort study undertook a comprehensive investigation to determine the effectiveness and feasibility of optimized drug treatment (ODT) for ATAAD. From January, 2024 to December, 2024, 572 ATAAD patients who were admitted to Beijing Anzhen Hospital and Beijing Dawanglu Emergency Rescue Hospital, 220 patients were surgically managed with open repair, 136 patients transferred to other hospitals for treatment, 216 patients were managed non-surgically because of severe comorbidities such as advanced age (> 70 years), malperfusion syndrome (defined as end organ damage diagnosed according to a combination of clinical symptoms and CT angiography showing dynamic flap obstruction) or patients’ wishes. After excluding 32 patients who died before starting any treatment, 184 patients were included in the final analysis (Additional file 1: Fig. S1).

The ODT strategy was based on current guidelines and the clinical experience of various medical specialists [1, 3]. It involves admitting the patient to the intensive care unit (ICU) immediately, conducting continuous invasive blood pressure monitoring, managing with adequate pain control, anti-impulse therapy (intravenous β-blockers, except in patients with contraindications), and strict blood pressure control (target systolic blood pressure < 120 mmHg, intravenous vasodilators should be used if the blood pressure is not well controlled after the initiation of intravenous β-blocker therapy) and controlling the heart rate (target 60–80 beats per minute) to prevent aortic dissection propagation and aortic rupture. Common medications used included esmolol infusion, intravenous labetalol, nicardipine, nitroprusside, and opiates. Owing to personal will and economic factors, patients who could not receive the ODT strategy were included in the non-ODT group. The non-ODT group of patients also received drug control for their blood pressure, heart rate, and other indicators, but without strict supervision by the guidelines. The details of data collection, definition of endpoint, follow-up information, and statistical analysis are shown in Additional file 1: Methods.

As shown in Additional file 1: Table S1, the ODT group included more patients with a history of transient ischemic attacks (TIA)/stroke, coronary heart disease, atrial fibrillation, gastrointestinal ulcer, diabetes, and hyperlipidemia. There was no significant difference in the history of hypertension between the two groups. More patients in the ODT group had organ malperfusion. Laboratory tests revealed that the ODT group had higher fibrinogen, total bilirubin, and direct bilirubin levels, as well as a lower albumin level, with no significant differences in other indicators. Under standardized medication supervision, more patients in the ODT group received β-blockers, vasoactive drugs, diuretics, α1 blockers, and angiotensin-converting enzyme inhibitors/angiotensin receptor blockers. The proportions of patients whose blood pressure and heart rate reached the standard were also greater in the ODT group (Additional file 1: Table S2). As shown in Additional file 1: Table S3, in the crude model, ODT was significantly associated with lower 30-day mortality (*HR* = 0.5, 95% CI 0.3–0.9, *P* = 0.010) and total mortality (*HR* = 0.6, 95% CI 0.4–0.9, *P* = 0.024). After adjusting for multiple potential covariates, the protective effect of ODT on 30-day mortality and total mortality remained significant (*HR* = 0.5, 95% CI 0.3–0.9, *P* = 0.019; *HR* = 0.6, 95% CI 0.4–1.0, *P* = 0.043, respectively). This significance persisted even after further adjustment for additional covariates (*HR* = 0.5, 95% CI 0.3–0.9, *P* = 0.019; *HR* = 0.6, 95% CI 0.3–1.0, *P* = 0.049, respectively).

Forest plots revealed that ODT decreased the risk of 30-day mortality and total mortality across various subgroups, regardless of age, sex, comorbidities, or left ventricular ejection fraction (Fig. 1a-b). All the patients completed their follow-up; 23 (44.2%) patients died in the ODT group, whereas 83 (62.9%) patients died in the non-ODT group. The Kaplan-Meier curves are shown in Fig. 1c. The mortality rate in the ODT group was significantly lower than that in the non-ODT group (log-rank, *P* = 0.023). Further landmark analysis (Fig. 1d) using 30 d as the cut-off point revealed that the mortality rate of patients in the ODT group was significantly lower than that in the non-ODT group (log-rank, *P* = 0.03), and there was no significant difference in the survival rate after 30 d (log-rank, *P* = 0.501).

**Fig. 1:**
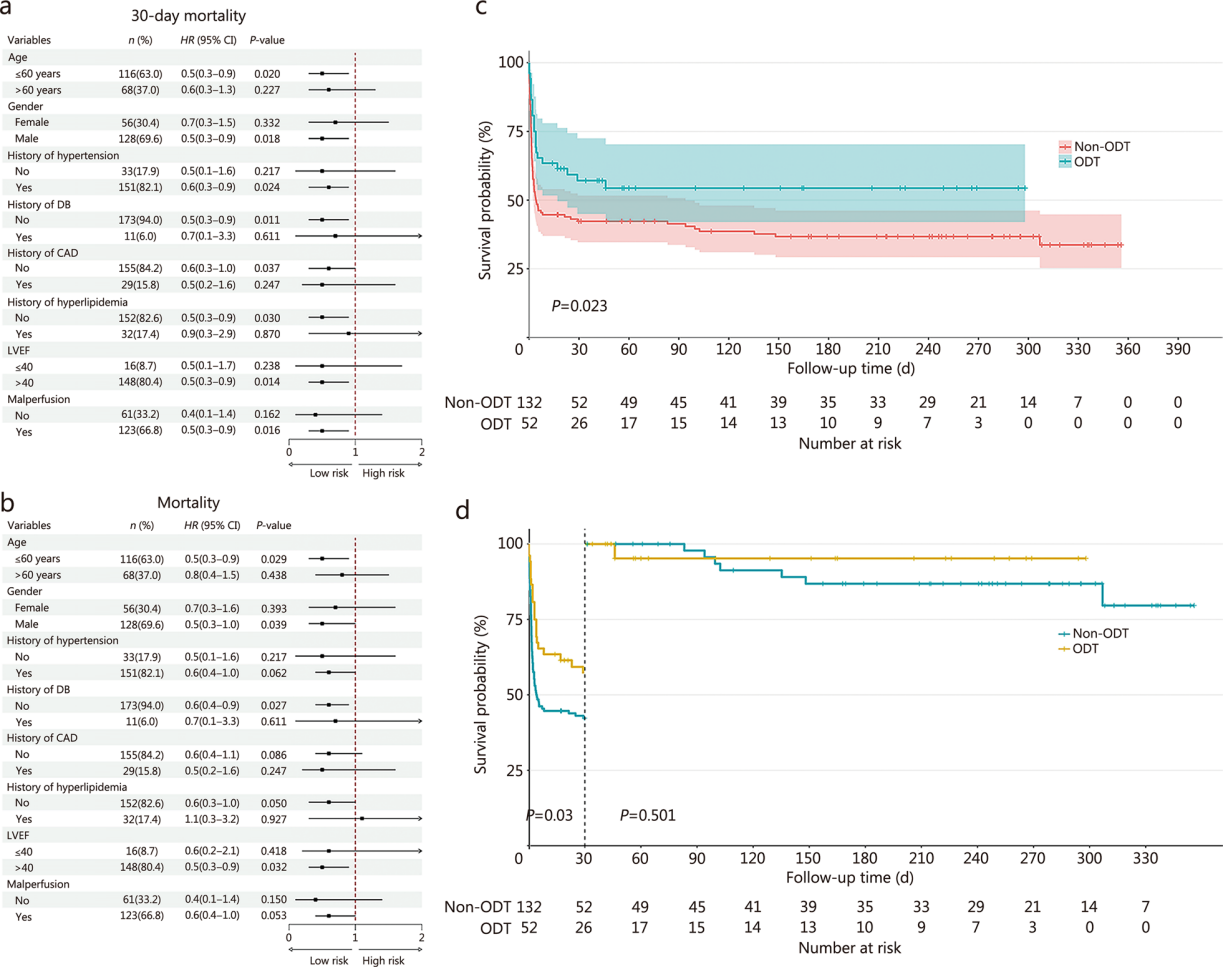
Impact of ODT on mortality evaluated by subgroup forest plots, Kaplan–Meier survival curves, and landmark analysis. **a** Forest plot for ODT and 30-day mortality based on different subgroups. **b** Forest plots for ODT and all-cause mortality based on different subgroups. **c** Kaplan–Meier survival analysis for the ODT and non-ODT groups. **d** Landmark analysis for the ODT and non-ODT groups. HR hazard ratio, CI confidence interval, ODT optimized drug treatment, DB diabetes, CAD coronary artery disease, LVEF left ventricular ejection fraction

Surgical treatment is still the first choice for ATAAD according to guidelines, but its high degree of difficulty and low penetration rate in primary hospitals pose challenges, especially in developing countries. According to the first large-scale aortic dissection registration cohort study in China, Sino-RAD, the rate of ATAAD surgery is only 52.6%, which is lower than 82.2% reported in the International Register of Aortic Dissection [4]. However, many centers still have surgery rates of less than 50% [5], and this number is extremely large in the overall population. Patients who do not undergo surgery often do not receive adequate medical assistance; thus, they are at constant risk of sudden death from aortic rupture, which further indicates that standardized and strict drug treatment should be placed in a more important position, even when there is no less than surgical treatment.

There are several limitations in our study. Firstly, the study is a retrospective observational cohort study with a relatively small sample size. Nevertheless, it should be noted that our study’s sample size remains one of the largest in the field of ATAAD conservative care. Secondly, there may be a bias in patient selection. A considerable number of patients who did not receive surgical treatment were due to non-medical factors, such as financial burden, inability to accept the risks of surgery, personal or family preferences, etc. Although we have made an effort to adjust for as many covariates as possible during the Cox regression analysis, it is possible that there were still covariates that had an impact on the results. A randomized, controlled trial may be worthwhile with strict inclusion and exclusion criteria.

In conclusion, ODT, as a noninvasive method, can significantly reduce mortality risk for ATAAD patients who refuse surgical treatment or lack surgical eligibility. In regions where resources are limited and surgery is not readily available, this treatment strategy should be promoted and strictly implemented to increase the compliance rate of the current guidelines. It is more convenient for medical institutions and more acceptable to patients and their families psychologically.

## Supplementary Information

**Additional file 1. **Methods. **Fig. S1** The flow chart of inclusion and exclusion criteria. **Table S1** Clinical characteristics of the ODT and non-ODT groups. **Table S2** Drug treatment strategies of the ODT and non-ODT groups.

## Data Availability

The datasets used or analyzed during the current study are available from the corresponding author upon reasonable request.

## Abbreviations

AAD: Acute aortic dissection;
ATAAD: Acute type A aortic dissection;
ODT: Optimized drug treatment
