# Supplementary material for: Clinical efficacy of optimized drug treatment for acute type A aortic dissection: insights from a multicenter retrospective cohort study
Source: Mil Med Res. 2025 Aug 22;12:52. doi: 10.1186/s40779-025-00638-8 (PMC12372315; doi:10.1186/s40779-025-00638-8)
Supplement: Supplementary file 1 — Additional file 1. Methods. Fig. S1 The flow chart of inclusion and exclusion criteria. Table S1 Clinical characteristics of the ODT and non-ODT groups. Table S2 Drug treatment strategies of the ODT and non-ODT groups. [file 40779_2025_638_MOESM1_ESM.pdf]

Methods

Data collection and endpoint

The clinical data encompassed various factors such as age, gender, the presence of diabetes (DB), hypertension, hyperlipidemia, transient ischemic attacks (TIA) or stroke, coronary heart disease, atrial fibrillation, and renal insufficiency. Additionally, the symptoms at the onset of acute type A aortic dissection (ATAAD), laboratory tests upon admission, and medication strategies during the treatment period were meticulously recorded. Moreover, the patients' discharge outcomes were carefully assembled from the Electronic Medical Records (EMR).

The primary endpoint of this study was all-cause mortality. 30-day mortality was defined as death occurring by any cause during the first 30 d after the onset.

Follow-up

After discharge, follow-up was conducted for all patients at 1 month, 6 months, and 12 months. Clinical follow-up and phone calls were used to obtain survival data. Patients who did not return for reexamination or lost contact were considered lost to follow-up. The changes in the condition were evaluated by reexamination of aortic CTA. This imaging modality provides crucial information about the status of the aorta and helps in determining any alterations in the patient's condition since discharge.

Statistical analysis

Normally distributed data were expressed as means ± standard deviation (SD), and differences between groups were analyzed using Student's *t*-test. Categorical variables were likewise analyzed by χ^2 or Fisher's exact tests.

Cox proportional hazard regression analysis was meticulously carried out to determine the independent predictive capacity of optimized drug treatment (ODT) on mortality. We present the regression results in the form of an unadjusted model as well as an adjusted model. In the subgroup analyses, we carried out stratification analyses with great care to examine whether the effects of ODT varied across diverse subgroups. This is demonstrated by a Forest plot. Additionally, we calculated the survival rate by means of the Kaplan-Meier method and landmark analysis. The between-group differences were evaluated using the log-rank test. Differences were regarded as statistically significant for $P < 0.05$. All the analyses were conducted with the statistical software packages R (http://www.R-project.org, The R Foundation).

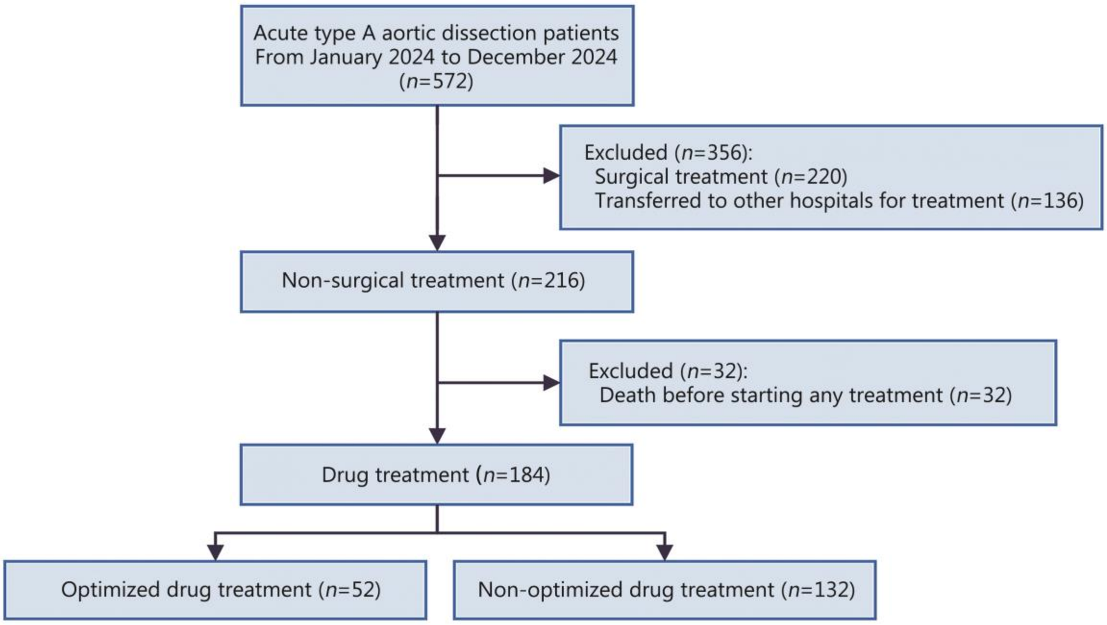

Fig. S1 The flowchart of the inclusion and exclusion criteria

Table S1 Comparison of clinical characteristics between ODT and non-ODT groups

**Variables**	**Total (*n* = 184)**	**Non-ODT (*n* = 132)**	**ODT (*n* = 52)**	***P*-value**
Age (mean ± SD)	55.6 ± 13.1	55.2 ± 12.8	56.8 ± 13.9	0.441
Gender [female, *n* (%)]	56 (30.4)	41 (31.1)	15 (28.8)	0.769
History of TIA/Stroke [*n* (%)]	14 (7.6)	5 (3.8)	9 (17.3)	0.002
History of CAD [*n* (%)]	29 (15.8)	15 (11.4)	14 (26.9)	0.009
History of AF [*n* (%)]	8 (4.3)	2 (1.5)	6 (11.5)	0.003
History of gastrointestinal ulcer [*n* (%)]	2 (1.1)	0 (0.0)	2 (3.8)	0.023
History of hypertension [*n* (%)]	151 (82.1)	107 (81.1)	44 (84.6)	0.571
History of DB [*n* (%)]	11 (6.0)	5 (3.8)	6 (11.5)	0.046
History of hyperlipidemia [*n* (%)]	32 (17.4)	13 (9.8)	19 (36.5)	< 0.001
SBP (mean ± SD)	137.2 ± 31.9	139.2 ± 32.5	132.1 ± 30.2	0.170
DBP (mean ± SD)	72.0 ± 19.5	71.1 ± 19.3	74.4 ± 20.0	0.295
HR (mean ± SD)	83.7 ± 20.0	82.6 ± 21.2	86.5 ± 16.6	0.238
Malperfusion [*n* (%)]	123 (66.8)	81 (61.4)	42 (80.8)	0.012
LVEF (mean ± SD)	56.3 ± 9.4	56.8 ± 8.7	54.8 ± 11.4	0.225
PT (mean ± SD)	12.8 ± 2.7	12.8 ± 2.4	12.8 ± 3.4	0.969
APTT (mean ± SD)	32.5 ± 15.4	33.6 ± 17.9	29.6 ± 4.1	0.109
FBG (mean ± SD)	3.1 ± 1.5	2.8 ± 1.3	3.8 ± 1.8	< 0.001
D-dimer (mean ± SD)	7413.4 ± 11793.3	7935.2 ± 12631.3	6088.8 ± 9313.5	0.340
CRP (mean ± SD)	46.0 ± 62.0	40.2 ± 61.8	58.3 ± 61.1	0.083
WBC (mean ± SD)	12.2 ± 4.7	12.4 ± 4.7	11.6 ± 4.6	0.301
Hb (mean ± SD)	127.4 ± 22.7	128.6 ± 22.9	124.6 ± 22.2	0.290
PLT (mean ± SD)	170.8 ± 67.1	166.0 ± 60.7	183.0 ± 80.5	0.121
CK (mean ± SD)	748.4 ± 4230.2	544.8 ± 1980.6	1336.5 ± 7667.4	0.280
LDH (mean ± SD)	469.6 ± 1451.9	522.4 ± 1688.9	326.6 ± 284.4	0.426
CKMB (mean ± SD)	10.5 ± 29.6	11.1 ± 32.3	9.0 ± 21.7	0.671
MYO (mean ± SD)	344.5 ± 768.7	375.0 ± 802.3	267.2 ± 677.2	0.393
ALT (mean ± SD)	91.8 ± 420.7	96.5 ± 470.4	79.7 ± 252.7	0.812
AST (mean ± SD)	255.5 ± 2195.0	321.6 ± 2585.6	87.7 ± 275.6	0.517

Variables	Total (n = 184)	Non-ODT (n = 132)	ODT (n = 52)	P-value
TP (mean ± SD)	64.2 ± 7.1	64.5 ± 7.3	63.6 ± 6.4	0.433
ALB (mean ± SD)	38.0 ± 4.8	38.5 ± 4.4	36.8 ± 5.6	0.039
TBIL (mean ± SD)	19.2 ± 10.8	18.1 ± 9.3	21.8 ± 13.7	0.043
DBIL (mean ± SD)	5.1 ± 4.5	4.5 ± 3.7	6.7 ± 5.9	0.004
Cr (mean ± SD)	165.7 ± 243.3	174.8 ± 256.6	142.7 ± 206.5	0.423
UA (mean ± SD)	411.2 ± 164.4	422.2 ± 161.0	381.9 ± 171.4	0.144
GLU (mean ± SD)	8.1 ± 3.2	8.2 ± 3.3	7.9 ± 2.6	0.553
Lac (mean ± SD)	2.3 ± 2.4	2.5 ± 2.7	1.9 ± 1.0	0.153

ODT optimized drug treatment, *TIA* transient ischemic attacks, *CAD* coronary artery disease, *AF* atrial fibrillation, *DB* diabetes, *SBP* systolic blood pressure, *DBP* diastolic blood pressure, *HR* heart rate, *LVEF* left ventricular ejection fraction, *PT* prothrombin time, *APTT* activated partial thromboplastin time, *FBG* fibrinogen, *CRP* C-reactive protein, *WBC* white blood cells, *Hb* hemoglobin, *PLT* platelets, *CK* creatine kinase, *LDH* lactate dehydrogenase, *CKMB* creatine kinase MB, *MYO* myoglobin, *ALT* alanine aminotransferase, *AST* aspartate aminotransferase, *TP* total protein, *ALB* albumin, *TBIL* total bilirubin, *DBIL* direct bilirubin, *Cr* creatinine, *UA* uric acid, *GLU* glucose, *Lac* lactic acid

Table S2 Comparison of drug treatment strategies between ODT and non-ODT groups [*n* (%)]

**Variables**	**Total (*n* = 184)**	**Non-ODT (*n* = 132)**	**ODT (*n* = 52)**	***P*-value**
β blockers	108 (58.7)	65 (49.2)	43 (82.7)	< 0.001
Vasoactive drugs	30 (16.3)	13 (9.8)	17 (32.7)	< 0.001
Diuretics	19 (10.3)	3 (2.3)	16 (30.8)	< 0.001
α1 blockers	74 (40.2)	47 (35.6)	27 (51.9)	0.042
CCB	84 (45.7)	61 (46.2)	23 (44.2)	0.808
Sodium nitroprusside	11 (6.0)	8 (6.1)	3 (5.8)	0.940
ACEI/ARB	6 (3.3)	0 (0.0)	6 (11.5)	< 0.001
Antiarrhythmic drugs	6 (3.3)	4 (3.0)	2 (3.8)	0.779
BP reaches standard	72 (39.1)	45 (34.1)	27 (51.9)	0.026
HR reach standard	82 (44.6)	48 (36.4)	34 (65.4)	< 0.001

CCB calcium channel blockers, *ACEI* angiotensin-converting enzyme inhibitors, *ARB* angiotensin receptor blockers, *BP* blood pressure, *HR* heart rate
